# Supplementary material for: Microbial Signatures Mapping of High and Normal Blood Glucose Participants in the Generation 100 Study
Source: Microorganisms. 2025 Nov 12;13(11):2582. doi: 10.3390/microorganisms13112582 (PMC12654564; doi:10.3390/microorganisms13112582)
Supplement: Supplementary file 1 [file microorganisms-13-02582-s001.zip › microorganisms-3931692-supplementary.pdf]

| Glucosose levels classification | mmol/L |
|---|---|
| Normal | 4-5.4 |
| Higher | >5.5 |

| | High glucose | Low glucose |
|---|---|---|
| Male | 8 | 22 |
| Female | 13 | 22 |
| BMI mean | 26.61484 | 25.51895 |
| BMI SD | 2.702073 | 3.497067 |
| CRP mean | 3.613 | 2.648864 |
| CRP SD | 7.346187 | 3.085679 |
| Glucose mean | 5.947619 | 5.013636 |
| Glucose SD | 0.676475 | 0.250201 |
| Visceral fat mean | 120.05 | 112.4091 |
| Visceral fat SD | 36.35421 | 31.22634 |

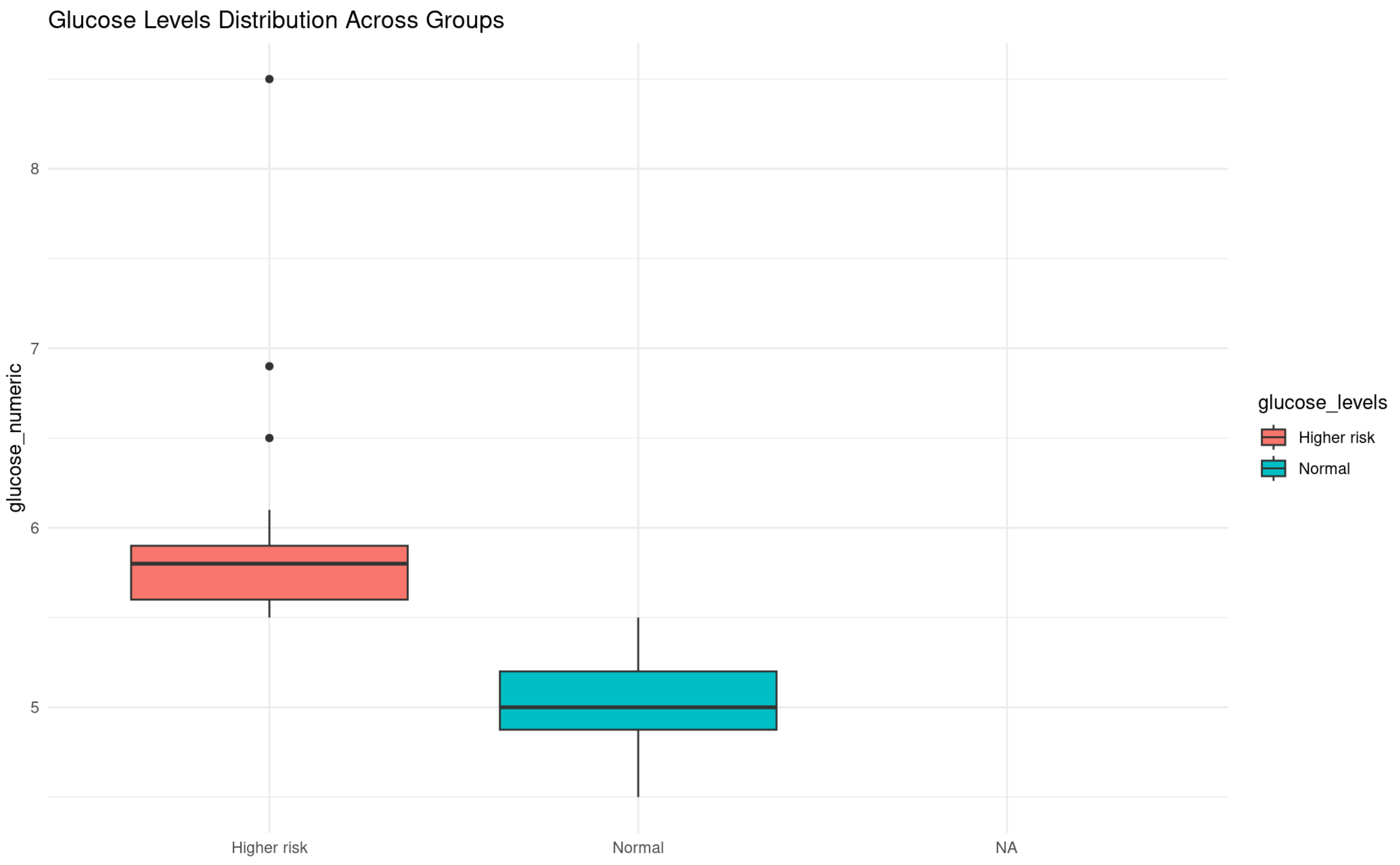


**Supplementary Figure S1.** Descriptive Statistics and glucose groups distribution.

| Descriptive stats | | | |
|---|---|---|---|
| Variable | Description / Levels | Summary | |
| Glucose status | Normal: 44 (67.7%)Higher risk: 21 (32.3%) | Mean fasting glucose = 5.32 ± 0.62 mmol/L(range: 4.5–8.5) | |
| Exercise (workout) group | Control: 28 (43.1%)HIIT: 27 (41.5%)MICT: 18 (27.7%) | 3 structured exercise arms | |
| Gender | Female: 39 (53.4%)Male: 34 (46.6%) | Nearly balanced | |
| BMI category | Healthy (31), Overweight (18), Underweight (6), Obese (18) | 42.5% healthy weight | |

**Supplementary Table S1.** Descriptive Statistics for the study population by variable

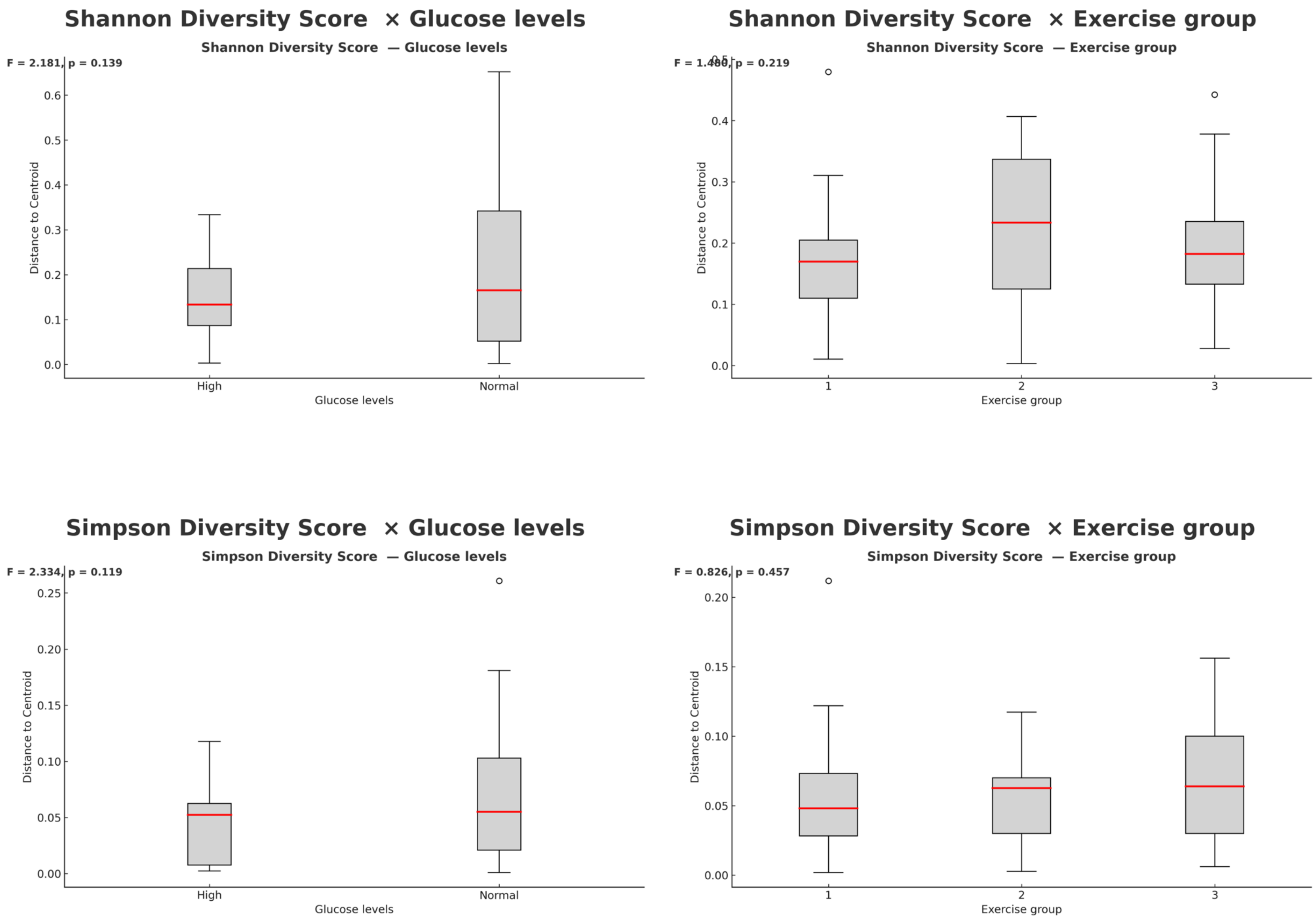


**Supplementary Figure S2.** Comparison of beta diversity dispersion and correlation between glucose levels and alpha diversity indices

**Supplementary Table S2.** Microbial composition at the phylum level. (*) marks significantly different abundance analysis

| | Glucose groups | | | Exercise groups | | |
|---|---|---|---|---|---|---|
| **Phylum** | **Higher (%) Group 2** | **Normal (%) Group 1** | **Normal ranges reported by Ortiz-Alvarez et.al 2020 (%)** | **Control** | **MICT** | **HIIT** |
| Firmicutes* | 62 | 57 | 60 to 65 | 59 | 51 | 59 |
| Bacteroidetes | 32 | 31 | 20 to 25 | 33 | 32 | 29 |
| Actinobacteria | 3 | 4 | 2 to 3 | 3 | 5 | 3 |
| Verrucomicrobia* | 2 | 5 | Variable | 3 | 8 | 3 |
| Proteobacteria | 2 | 3 | 5 to 10 | 2 | 3 | 5 |

* FDR p-values <0.05

**Supplementary Table S3.** Differential abundance analysis at the phylum level between normal glucose and high glucose

**Differential Abundance Analysis at the Phylum level (Normal glucose versus high glucose)**

| Name | Max group mean | $Log_2$ fold change | Fold change | P-value | FDR p-value |
|---|---|---|---|---|---|
| Synergistetes | 46.59 | 5.05 | 33.24 | 0.00000109* | 0.000013* |
| Elusimicrobia | 2.8 | 3.98 | 15.73 | 0.000984* | 0.0059* |
| Euryarchaeota | 1,055.73 | 2.24 | 4.74 | 0.00675* | 0.03* |
| Verrucomicrobia | 5,352.18 | 1.4 | 2.64 | 0.01* | 0.04* |
| Proteobacteria | 3,137.32 | 0.93 | 1.91 | 0.04 | 0.09 |
| Firmicutes | 59,561.00 | -0.15 | -1.11 | 0.23 | 0.47 |
| Actinobacteria | 3,674.66 | 0.22 | 1.16 | 0.52 | 0.77 |
| Fusobacteria | 44.57 | 0.76 | 1.69 | 0.49 | 0.77 |
| Bacteroidetes | 31,009.27 | -0.04 | -1.03 | 0.81 | 0.94 |
| Cyanobacteria | 13.14 | -0.12 | -1.09 | 0.86 | 0.94 |
| TM7 | 12.2 | 0.15 | 1.11 | 0.79 | 0.94 |
| Tenericutes | 353.73 | -0.03 | -1.02 | 0.96 | 0.96 |
